# Development and Real‐Time Clinical Application of New Transcription‐Less Discourse Assessment Approaches for Arabic Speakers With Aphasia

**DOI:** 10.1111/1460-6984.70043

**Published:** 2025-05-05

**Authors:** Reem S. W. Alyahya

**Affiliations:** ^1^ Department of Language and Communication Sciences, School of Health and Psychological Sciences, City University of London London UK; ^2^ Communication and Swallowing Disorders Department King Fahad Medical City Riyadh Saudi Arabia

**Keywords:** aphasia, Arabic, assessment, checklists, content word fluency, discourse, fluency, main concepts

## Abstract

**Background:**

Assessing spoken discourse during aphasia clinical examination is crucial for diagnostic and rehabilitation purposes. Recent approaches have been developed to quantify content word fluency (CWF) and informativeness of spoken discourse without the need to perform time‐consuming transcription and coding. However, the accuracy of these approaches has not been examined in real‐time clinical settings, and they have been developed and validated mainly in English and thus cannot be applied to other languages.

**Aims:**

For the first time: (i) to create and validate CWF checklists and main concept (MC) lists in Arabic; (ii) to examine the application of these two approaches in real‐time clinical settings with people with aphasia (PWA) while they are performing the task; and (iii) to investigate whether these two approaches can differentiate discourse responses produced by PWA versus neurotypical adults.

**Methods:**

The Arabic Discourse Assessment Tool (ADAT) was used to collect discourse responses on three tasks (composite picture description, storytelling narrative, and procedural discourse) from 70 neurotypical control adults and 50 PWA matched to the control group in age and education. The discourse samples were transcribed, and analysed. For each task, CWF checklists and MC lists were developed and validated using discourse reponses from the control group. Afterwards, the application of these two approaches in real‐time clinical settings was examined with the aphasia groups. The psychometric properties of CWF and MC approches were examined.

**Results:**

Novel Arabic CWF checklists and MC lists were successfully developed, validated, and applied clinically for three discourse tasks. The analysis showed significant high accuracy between CWF scores obtained in real‐time clinical settings and those identified using the traditional approach of transcribing and analysing discourse samples across all three discourse tasks (ICC = 0.88 to 0.94). Furthermore, significant excellent reliability (*ICC* = 0.917 to 0.994) were found for the three tasks in both groups. Most of the MCs were produced accurately and completely by neurotypical control adults, whereas they were mostly absent in PWA. CWF checklists and MC lists showed significant high validity in distinguishing spoken discourse produced by PWA from those produced by neurotypical adults at *p* < 0.001.

**Conclusions:**

This is the first study to develop and validate novel Arabic CWF checklists and MC lists. Furthermore, this study demonstrated that the CWF approach and main concept analysis (MCA) can be applied clinically in real‐time with PWA. These transcription‐less approaches can be used as part of the routine aphasia clinical examination to provide quick but accurate assessments of CWF (microlinguistics) and informativeness (macrolinguistics) of spoken discourse in PWA. These approaches also provide a significant resource for Arabic speakers with aphasia, that will lead to accurate aphasia assessments and better clinical management.

**WHAT THIS PAPER ADDS:**

*What is already known on the subject*

- Conversations between people rely heavily on producing relevant and informative spoken discourse. Thus, there has been increased interest in the application of discourse tasks in clinical practice to enable a comprehensive assessment of expressive language and functional communication. However, utilising discourse tasks in clinical settings is impractical because their administration and scoring are time‐consuming, labour‐heavy, and must be completed after the session has ended. Therefore, transcription‐less, efficient, and accurate approaches to analyse discourse responses have been developed and validated in English. However, no study has investigated the application of these approaches in real‐time clinical settings with people with aphasia (PWA).

*What this study adds*

- To our knowledge, this is the first study to (i) develop and validate content word fluency (CWF) checklists and main concept (MC) lists in Arabic. These were created for three discourse tasks (composite picture description, storytelling narratives, and procedural discourse) using discourse responses from a neurotypical control group; and (ii) examine the application of these valid, transcription‐less, efficient, and accurate approaches to assess CWF and informativeness of spoken discourse in PWA in real‐time clinical settings.

*What are the potential or actual clinical implications of this work?*

- The transcription‐less, efficient, and accurate discourse assessment approaches that have been developed and validated in this study allow clinicians to objectively assess discourse responses produced by Arabic speakers with aphasia during clinical examinations while the person is speaking without the need for offline transcription, analyses, and scoring. The reliable application of these approaches in real‐time clinical settings encourages clinicians to incorporate discourse tasks as part of their routine clinical examination with PWA to be used (i) for a comprehensive assessment of expressive language skills beyond words and sentences, (ii) to guide the development of personalised therapy goals and strategies to improve functional communication, and (iii) as outcome measurements to monitor spontaneous recovery and changes in response to interventions.

## 1 Introduction

Conversation between people relies heavily on producing meaningful spoken discourse. This skill, however, is invariably impaired to some degree in people with aphasia (PWA). As a result, PWA experience reduced engagement in conversation and diminished communication with others, which limits social participation and restricts activities of daily living (Corsten et al. 2014).

Discourse is defined as language beyond sentence and phrase levels, which is used to convey messages in a meaningful and interactive way (Bryant et al. 2016). Producing discourse is highly demanding, as several complex linguistic and cognitive processes need to be engaged to produce an accurate and meaningful message. Generally, two interconnected processes support the production of discourse: microlinguistics, which includes lexical, grammatical, and syntactic processing, and macrolinguistics, which are concerned with the organisation and maintenance of appropriate and meaningful concepts (Alyahya 2024b; E. Armstrong 2000).

### 1.1 Assessment of Spoken Discourse

Discourse deficits are highly prevalent in PWA (Alyahya et al. 2020; Alyahya et al. 2022; Stark 2019). Clinical assessments of language impairments in aphasia are conducted using standardised assessment tools. However, published aphasia assessment tools have low ecological validity, and scores on these tools do not reflect naturalistic language and may not predict real‐time performance (Fergadiotis and Wright 2015; Marini et al. 2011). On the other hand, analysing spoken discourse provides significant insights into various elements of expressive language (Bryant et al. 2016; Dipper and Pritchard 2017) and might provide a more sensitive evaluation of communication skills than existing standardised aphasia assessment tools. A range of tasks can be employed to elicit and assess spoken discourse, including describing a static scene, narrating a story, and providing a step‐by‐step description of a procedure (Bryant et al. 2016). During routine clinical examination, picture description is commonly used to assess spoken discourse, and it is a subtest in all major aphasia assessment tools, such as the Western Aphasia Battery (Kertesz 1982) and the Comprehensive Aphasia Test (Swinburn et al. 2005). However, there are several limitations to existing discourse assessment tools. Firstly, the gold standard approach used to assess spoken discourse is through collecting and analysing discourse samples. However, the collection and offline transcription and analysis of discourse samples is extremely effortful and time‐consuming, making its utilisation impractical in clinical settings. Additionally, the lack of an objective approach makes it potentially influenced by subjective judgment. A survey of speech‐language pathologists/therapists (SLTs) revealed that only 6% of clinicians always use discourse analysis as part of their clinical examination (Cruice et al. 2020). Time constraints, lack of resources, and examiner confidence were reported to be the main barriers (Bryant et al. 2016; Cruice et al. 2020). It has been estimated that one minute of discourse could take up to one full hour to transcribe and analyse, depending on aphasia severity and the amount of analysis (L. Armstrong et al. 2007). These time constraints might prevent the examination of language deficits at higher levels and the assessment of how the interaction between language domains can affect functional communication. Second, for a discourse approach to be deemed clinically viable, it is essential to show that the approach can be used in real‐time clinical settings with patients, rather than just being retrospectively used on recorded discourse samples collected from research participants. A striking finding from recent reviews showed that there has been no published evidence of these transcription‐less discourse approaches being used with PWA in clinical settings, despite their large application in research (Bryant et al. 2016; Stark et al. 2021). Third, published aphasia assessment tools tend to be biased towards English and European languages and Western cultures. There has been a recent increased awareness and interest in developing aphasia assessment resources in non‐English languages, including Arabic (Altaib et al. 2021; Alyahya and Druks 2016). Arabic is the fourth most spoken language worldwide, with over 280 million native Arabic speakers. Due to the lack of comprehensive Arabic aphasia assessment tools, SLTs in Gulf countries tend to rely on informal assessments or the use of non‐adapted English assessment tools (Khoja 2019). Informal assessments depend on subjective judgement and can lead to inaccurate diagnoses. The use of Western English aphasia assessment tools to examine language impairment with Arabic speakers is also problematic due to linguistic and cultural differences.

This highlights the need to develop aphasia assessment resources that are linguistically and culturally suitable for use by Arabic speakers. Furthermore, researchers and clinicians have highlighted the importance of developing fast and efficient approaches to analyse spoken discourse, ideally without the need for transcription, in order to be used in clinical practice (L. Armstrong et al. 2007; Kim et al. 2019) and as part of a core outcome set of discourse measures (Dietz and Boyle 2017).

### 1.2 Transcription‐Less Discourse Assessment Approaches

Transcription‐less approaches to discourse analysis would make assessments of spoken discourse accessible to SLTs and useable as diagnostic and outcome measurements in clinical and research settings (L. Armstrong et al. 2007). These approaches should be sufficiently simple, ecologically valid, and truly represent the speaker's discourse skills to be used in clinical and research settings as assessment and outcome measures and for comparability purposes (E. Armstrong 2018). Few clinician‐friendly transcription‐less approaches to analyse discourse responses have been developed, including core‐lexicon (Dalton and Richardson 2015; Kim et al. 2023, 2019; MacWhinney et al. 2011) and content word fluency (CWF) approach (Alyahya et al. 2022). Core‐lexicon is a lexicon‐based measure of word retrieval in discourse, while CWF measures the fluency of content words during spoken discourse. Both measures do not require collecting or transcribing discourse samples and are based on normal expectations of discourse production using pre‐specified checklists of items created from discourse responses produced by healthy controls. The checklists are used to objectively examine whether these items are in the active vocabulary of the speaker, and CWF further assesses the fluent production of these items. The development and scoring of these checklists differ between the two approaches. Core‐lexicon involves assigning one point to each lexical item, regardless of how often the word was produced. On the other hand, CWF involves assigning a point each time a word from the pre‐specified checklist is produced, to index CWF. CWF is a data‐driven approach that involves developing and validating checklists from an independent group of neurotypical control adults (Alyahya et al. 2022). CWF are potentially clinically viable as they are easy to administer, and do not require offline transcription or analyses. It has been shown that these clinician‐friendly transcription‐less approaches provide better reflection of content word production compared to picture naming at the chronic phase in a large group of 48 PWA with a wide range of aphasia severities and classifications (Alyahya et al. 2022); and in a longitudinal study with 19 PWA (Kim et al. 2023). Studies have shown that these transcription‐less approaches can differentiate the performance of PWA from healthy controls (Alyahya et al. 2022; Dalton and Richardson 2015), and fluent from non‐fluent aphasia (Kim et al. 2019). Furthermore, strong correlations have been shown between these transcription approaches and discourse macrolinguistic features (e.g., informativeness and main concepts [MCs]) in large studies with 48 PWA with a wide range of aphasia severities and classifications (Alyahya et al. 2022), and a large dataset of 238 PWA (Dalton and Richardson 2015).

Main concept analysis (MCA) is another discourse‐level approach that has been used to measure the content of discourse responses in aphasia (Nicholas and Brookshire 1993, 1995). This is done to assess the speaker's ability to produce accurate and complete essential information of a discourse using a predetermined list of key concepts (Nicholas and Brookshire 1993, 1995). This approach applies a multilevel coding system on a list of MCs, which correspond to a closed set of utterances where each utterance consists of a subject, one main verb, and any subordinate clauses, which are used to reflect the gist of the discourse (van Dljk and Kintsch 1983). MCA provides important information about both microlinguistic and macrolinguistic discourse structure and correlates with subtests from the Cantonese version of the Western Aphasia Battery (spontaneous speech, fluency, and naming scores) (Kong 2009; Yiu 1992). MCAs are stimulus‐dependent, and they have been developed for several discourse tasks in English (Dalton and Richardson 2015; Nicholas and Brookshire 1993, 1995; Richardson and Dalton 2016, 2020) and Cantonese (Kong 2009).

One of the strengths of CWF and MCA is the checklist format, which has the potential to allow clinicians to simply check off the items on the checklist online during clinical examinations without the need for offline transcription and analyses. The psychometric properties of the two approaches are strong, they are ecologically valid, with high test–retest and inter‐rater reliability. These approaches can discriminate between neurotypical and clinical populations and has the potential to monitor intervention effectiveness (Alyahya et al. 2022; Dalton and Richardson 2015; Dipper and Pritchard 2017; Kong 2009; Richardson and Dalton 2016). However, previous aphasiology studies that used transcription‐less approaches to assess discourse deficits applied these approaches offline through listening to the recorded discourse sample produced by research participants, rather than using them online during clinical examination while the patient is speaking (Alyahya et al. 2022; Dalton and Richardson 2015; Kim et al. 2019; Nicholas and Brookshire 1993, 1995; Richardson and Dalton 2016, 2020).

### 1.3 The Present Study

The purpose of this study was to develop and validate novel CWF checklists and MC lists for the three discourse stimuli of the Arabic Discourse Assessment Tool (ADAT) (Alyahya 2024a) from discourse responses produced by neurotypical control Arabic speakers. Subsequently and for the first time, the study aimed to apply and examine these transcription‐less approaches in real‐time clinical settings and assess deficits in spoken discourse in PWA. Finally, this study investigated whether these two approaches can differentiate discourse responses produced by PWA from those produced by neurotypical adults, which was used to assess the construct validity of the two approaches (Streiner and Norman 2000).

## 2 Methods

This study was approved by King Fahad Medical City's Institutional Review Board (IRB No. 20–763).

### 2.1 Participants

A large aphasia group and an age‐ and education‐matched neurotypical control group took part in this study. The inclusion criteria for all participants were adults above the age of 18 years old, native Arabic speakers, with self‐reported normal and/or corrected‐to‐normal hearing/vision. Further inclusion criteria for participants in the aphasia group included being diagnosed with aphasia following brain damage and being medically and neurologically stable at the time of participation (i.e., at least 1 month post onset). Neurological and medical histories were obtained from the patient's medical records as documented by their neurologists. The exclusion criterion for the control group was self‐reported previous history of neurological conditions or brain damage/injury. The exclusion criterion for the aphasia group was a history of developmental speech or language difficulties before the onset of brain damage.

A total of 70 neurotypical control adults (34 males) and 50 PWA (32 males) participated in this study. Demographic information for both groups is reported in Table 1. Aphasia was confirmed by the author using the Short Aphasia Test for Gulf Arabic speakers (SATG) (Altaib et al. 2021). In order to be able to provide a discourse sample, non‐verbal participants (i.e., who could not produce any spoken words on the SATG) were excluded from this study. All participants were recruited from King Fahad Medical City in Riyadh, Saudi Arabia, and provided written informed consent before voluntarily participating in this study according to the Declaration of Helsinki under the approval of the local research ethics committee.

**TABLE 1:** Participants’ demographic and medical information.

	Control group (*N* = 70)			Aphasia group (*N* = 50)		
	Mean	SD	Range	Mean	SD	Range
**Age (years)**	48.24	9	29–74	50.6	16.3	18–86
**Education (years)**	12.37	5	0–18	11.1	4.95	0–22
**Months post onset**	N/A			16.8	24.5	1–105
**Medical diagnosis**	N/A			Stroke = 42		
				Traumatic Brain Injury = 5		
				Tumour resection = 2		
				Viral encephalopathy = 1		

### 2.2 Discourse Tasks

The ADAT was used to elicit discourse responses (Alyahya 2024a). This tool consists of three discourse stimuli (composite picture description, storytelling narrative, and procedural discourse) that are culturally and linguistically appropriate to Arabic speakers (Alyahya 2024a). The first task employed a picture‐supported stimulus called the ‘Lounge composite picture description’, which involved a scene of multiple characters and events, and was used to elicit descriptive discourse. The second task used a picture‐supported stimulus called the ‘Kitchen storyboard’, which was used to elicit a narrative discourse. The third task elicited procedural discourse by asking participants ‘how they prepare a cup of tea’. The three stimuli were used to collect discourse samples from all participants: first the neurotypical control group and then PWA.

### 2.3 Study Procedure

Each participant attended a single session. The three discourse tasks were administered in a randomised order. Picture‐supported stimuli were displayed, and participants were instructed to describe what is going on in the picture in as much detail as possible. They were allowed to look through the picture before describing it. For the three discourse samples, no time limit was imposed during responses, and no minimum number of words produced was required for each discourse sample. No prompts were provided by the examiner throughout testing. Responses were continuously audio recorded for offline transcription and analysis. For the aphasia group, CWF checklists were also computed in real‐time during the session (i.e., without having to listen to the audio recording or referring to the transcripts).

### 2.4 Discourse Transcription and Analysis

Each discourse sample was transcribed verbatim (orthographically) by trained SLT assistants. Twenty‐five percent of the samples were randomly selected and transcribed by another transcriber. Transcription agreement coefficient was calculated for each transcript using point‐by‐point percent agreement to determine the degree of transcription accuracy for each discourse task. The analyses revealed excellent transcription accuracy on the three discourse tasks in both groups: (i) neurotypical control group: 91% agreement for descriptive discourse, 92% agreement for storytelling narrative, and 95% agreement for procedural discourse; (ii) aphasia group: 92% agreement for descriptive discourse, 92% agreement for storytelling narrative, and 95% agreement for procedural discourse.

Subsequently, percent correct information units (CIU) was extracted from each transcript. CIU is the sum of all intelligible, accurate, reliable, and relevant words—including words in incomplete utterances and those used in a grammatically incorrect form (Nicholas and Brookshire 1993). CIU was calculated in this study because it has strong psychometric properties of construct validity and reliability, good ecological validity, high diagnostic sensitivity even in people with mild aphasia, and it is sensitive to detect changes in connected speech over time across different types of discourse elicitation techniques (Dipper and Pritchard 2017; Marini et al. 2011; Nicholas and Brookshire 1993). Twenty‐five percent of the transcripts were randomly selected and analysed by another trained rater to examine inter‐rater reliability using intraclass correlation coefficient (ICC) with a two‐way mixed effects model. The analyses revealed significant excellent inter‐rater reliability (*p* < 0.001) on the three discourse tasks in both groups: (i) neurotypical control group: ICC = 0.954 for descriptive discourse, ICC = 0.917 for storytelling narrative, and ICC = 0.956 for procedural discourse; (ii) aphasia group: ICC = 0.991 for descriptive discourse, ICC = 0.988 for storytelling narrative, and ICC = 0.994 for procedural discourse.

Furthermore, the discourse samples collected from the neurotypical control group were analysed to create Arabic CWF checklists and MC lists. The discourse transcripts from both groups were analysed to examine the reliability and validity of the two approaches as described below.

### 2.5 The Development and Psychometric Examination of CWF Approach

CWF is a transcription‐less, efficient, and accurate approach to measure content word production during spoken discourse, which has been developed and validated for two pictorial stimuli in English (Alyahya et al. 2022). To adapt this approach to Arabic, the same protocol used in the original paper (Alyahya et al. 2022) was replicated here on different discourse stimuli from ADAT, and responses produced by Arabic speakers. The protocol is described below.

To provide an estimate of out‐of‐sample prediction accuracy, Arabic CWF checklists were designed and tested for each of the three discourse stimuli using the discourse samples produced by the neurotypical control group—an entirely separate dataset from the PWA's dataset. The checklists were derived to reflect the fluent production of content words, irrespective of the word class. Specifically, content words that were most commonly/consistently produced by the majority of neurotypical participants (i.e., ≥ 75% of the participants) were identified through rank ordering the produced words in terms of frequency. Hence, all lexical items within the checklists are accurate and relevant to the discourse topic. After creating the checklists, a total score on each checklist was computed for each participant as follows: every time the participant produced one word from the target checklist items, a point was given, and the total count increased. This included when the target words were used again in subsequent phrases but excluded immediate repetitions and perseverations. Criterion validity of the CWF approach for each of the three checklists was examined using Pearson's correlation coefficient to determine the similarity between CWF scores and CIU values extracted using the standard approach of fully transcribing and analysing the discourse samples. The CWF checklists and scoring sheets are available in Supporting Information.

### 2.6 The Development of MC Lists

MCA is a content‐based discourse analytic approach used to quantify the presence of essential discourse elements, the accuracy of the provided information, and the completeness of essential information in spoken discourse (Nicholas and Brookshire 1993, 1995). An MC is a relevant statement that consists of one main verb, constituent arguments, and associated subordinate clauses and/or prepositional phrases, and it must outline the gist/essential information of the discourse. To adapt this measure to Arabic, the original procedure (Nicholas and Brookshire 1993, 1995) was replicated here on different discourse stimuli and responses produced by Arabic speakers. In this study, Arabic MC lists were created from the neurotypical controls’ discourse data in three stages, and then they were tested in the aphasia group. The procedure is described below.

Three MC lists were created for each of the ADAT's discourse stimuli using the neurotypical controls’ discourse data to reflect the essential concepts of the discourse. Specifically, three stages were followed for each discourse stimulus. In the first stage, potential concepts were created by 12 Arabic speakers trained on creating concept statements. They wrote a list of concepts, where each concept consisted of a statement that described an essential event of the discourse and was a full sentence that included one verb and its arguments. Concepts that were written by half or more of the speakers were entered into lists of potential concepts to be used in the next stage. In the second stage, a trained research assistant identified the presence of the potential concepts in each transcript. Afterwards, final MC lists were created by the author, which consisted of MCs that were produced by 70% of the neurotypical adults. This process was repeated for the three discourse stimuli. Each MC consisted of a verb, essential elements (all words that were produced by 70% of the neurotypical adults), and non‐essential elements that were used to aid in the contextualization of the MC. Non‐essential elements were not included in the scoring process. The MC have commonly acceptable alternative lexical items that do not change the meaning of the concept, which were derived from the neurotypical controls’ data. The third stage involved scoring each MC from the neurotypical controls’ data using an established multilevel coding system (Kong 2009; Nicholas and Brookshire 1995; Richardson and Dalton 2016). Trained research assistants who were blinded to the first and second stages coded the data by assigning one of the following five codes to each MC: AC (accurate and complete concept: the speaker produced all essential elements of the MC correctly); AI (accurate but incomplete concept: the essential elements of the MC were partially produced by the speaker but they were correctly produced, in that at least one essential element of the MC was omitted); IC (inaccurate but complete concept: the speaker produced all essential elements, but at least one essential element was inaccurate); II (inaccurate and incomplete concept: the speaker produced at least one essential element incorrectly, plus they omitted at least another essential element); and AB (absent concept: the concept was not produced by the speaker). Generally, complete concept means all essential elements were produced by the speaker, and accurate concept means the speaker correctly produced the essential elements. After coding all concepts, an a priori formula was used to generate an MC score for each participant on the three discourse stimuli, as follows:

(#AC×3)+(#AI×2)+(#IC×2)+(#II×1).

### 2.7 Applications of CWF and MCA in PWA

After creating novel CWF checklists on the three discourse stimuli of ADAT, the checklists were tested with PWA in real‐time during clinical examination. Inter‐rater reliability was examined using ICC. To examine the criterion validity of this approach, Pearson's correlation coefficients were calculated between CWF scores and CIU values extracted using the traditional approach of fully transcribing and coding each discourse sample. To further assess the accuracy of the real‐time clinical application of CWF checklists compared to the more laborious task of transcribing and analysing discourse samples, CWF scores measured in real‐time were compared to CWF scores identified from the transcripts using ICC with a two‐way mixed effects model.

After creating MC lists on the three discourse stimuli, MCA was carried out on the discourse responses produced by PWA to analyse for the presence, accuracy, and completeness of essential discourse information using the same multilevel coding system that was used with the neurotypical adult group (Kong 2009; Nicholas and Brookshire 1995; Richardson and Dalton 2016). The MC lists and scoring sheets are available in Supporting Information.

### 2.8 Comparison Between Neurotypical Control and Aphasia Groups

Cut‐off levels of impairment on the two approaches (CWF and MCA) for each discourse task were established using the neurotypical control data, in that impairment was defined as scoring 1.5 SD below the mean of the neurotypical control group, as recommended by Brooks et al. (2011). Composite scores were established for CWF and MCA by summing across scores on all three discourse tasks for each participant. To examine the construct validity of the two approaches, the performance of the aphasia group was compared to the performance of the neurotypical control group using one‐way ANOVA with group as the between‐subject factor and Bonferroni correction for multiple comparisons.

## 3 Results

### 3.1 New Arabic CWF Checklists

Three novel CWF checklists were successfully developed and derived from the discourse responses produced by the neurotypical control group, as follows: (i) 11 target items for the ‘Lounge picture description’; (ii) 10 target items for the ‘Kitchen storytelling narrative’; and (iii) 14 target items for the ‘tea—procedural discourse’, in addition to their acceptable alternative lexical items. Arabic CWF checklists and scoring sheets are provided in Supporting Information. Criterion validity analyses showed significant moderate‐to‐high correlations between CWF score and CIU values in the neurotypical control group: picture description *r* = 0.67; storytelling narrative *r* = 0.69; and procedural discourse *r* = 0.88 (two‐tailed *p* < 0.001).

### 3.2 New Arabic MC Lists

Three novel Arabic MC lists were successfully developed and derived from the neurotypical controls’ discourse data, as follows: (i) 4 MCs for the ‘Lounge picture description’, with a maximum score of 12 based on the formula provided in the Methods; (ii) 3 MCs for the ‘Kitchen storytelling narrative’, with a maximum score of 9; and (iii) 4 MCs for the ‘tea—procedural discourse’, with a maximum score of 12. Thus, the maximum MC composite score is 33. The Arabic MC lists and scoring sheet are provided in Supporting Information. All MCs of the ‘Lounge picture description’ were produced by 89.86% of the neurotypical control speakers, the MCs of the ‘Kitchen storytelling narrative’ were produced by 86.96% of the neurotypical control speakers, and the MCs for the ‘tea—procedural discourse’ were produced by 93.94% of the neurotypical control speakers. The vast majority of the MCs received a coding of ‘accurate and complete’ by the majority of the neurotypical adult speakers across all three discourse tasks (details are reported in Table 2).

**TABLE 2:** Scoring of main concepts for different discourse tasks in neurotypical controls and PWA.

	Neurotypical adult group (*N* = 70)			Aphasia group (*N* = 50)		
MC coding	Picture description	Storytelling narrative	Procedural discourse	Picture description	Storytelling narrative	Procedural discourse
**AC**	55.80	54.11	70.83	30	16.00	35.5
**AI**	16.67	22.71	10.23	15	14.00	18.5
**IC**	10.51	5.31	2.27	6.5	1.33	2
**II**	6.88	4.83	10.61	13.5	8.00	8.5
**A**	10.14	13.04	6.06	35	60.67	35.5

*Note*: Values are percentages.

Abbreviations: AC = accurate/complete, AI = accurate/incomplete, IC = inaccurate/complete, II = inaccurate/incomplete, A = absent.

### 3.3 Real‐Time Clinical Application of CWF and MC in Aphasia

**FIGURE 1:**
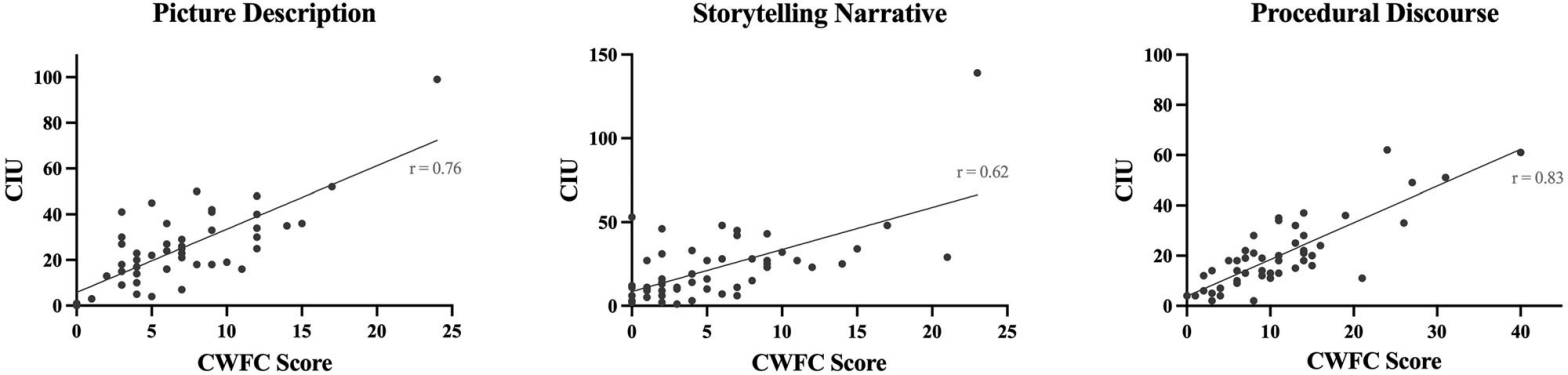
The relationship between measures of content word production during spoken discourse in aphasia. Scatterplots illustrating the correlations between scores on the transcription‐less CWF approach (*x* axis) and CIU values extracted using the traditional transcription and quantitative scoring approach (*y* axis) across different discourse stimuli in people with aphasia (*N* = 50). All correlations are significant at *p* < 0.001.

The CWF checklists were successfully applied in real‐time during clinical examination with PWA. The criterion validity analysis revealed significant moderate‐to‐high correlations between CWF scores and CIU values in PWA: picture description *r* = 0.76, storytelling narrative *r* = 0.62, and procedural discourse *r* = 0.83 (two‐tailed *p* < 0.001). These correlations are illustrated in Figure 1. Furthermore, ICC analyses revealed significant very good to excellent agreement between CWF scores obtained in real‐time versus those identified through the traditional laborious transcription and analysis of discourse samples for all three discourse stimuli: ICC = 0.94 for descriptive discourse, ICC = 0.83 for storytelling narrative, and ICC = 0.88 for procedural discourse (*p* < 0.1001). These results indicate high validity and reliability of the real‐time clinical application of the CWF approach.

As opposed to neurotypical control adults, the vast majority of MCs received a coding of ‘absent’ by the majority of PWA across all three discourse tasks (details are reported in Table 2).

### 3.4 Comparison Between the Neurotypical Control Group and the Aphasia Group

Descriptive statistics and normative data on the discourse measures and new approaches used in this study for both groups are reported in Table 3. Impairment cut‐offs were established for CWF and MCA on each discourse task using the norms as described in the Methods. Results are presented in Table 3. The findings indicated that the majority of PWA were impaired on both CWF and MCA, as follows: (i) during descriptive discourse: 66% of PWA were impaired on CWF, and 74% were impaired on MCA; (ii) during narrative discourse: 64% of PWA were impaired on CWF, and 88% were impaired on MCA; and (iii) during procedural discourse: 40% of PWA were impaired on CWF, and 66% were impaired on MCA.

**TABLE 3:** Normative data and descriptive statistics of discourse measures in neurotypical controls and people with aphasia.

		Neurotypical adult group (*N* = 70)				Aphasia group (*N* = 50)		
Discourse Task	Discourse measure/approach	Mean	SD	Range	Cut‐off^*^	Mean	SD	Range
**Picture description**	Token counts	49.56	18.75	15–94	—	47.46	29.64	6–151
	Duration	40.57	17.22	12–101	—	91.04	69.60	28–322
	%CIU	81.92	11.71	55.36–100	65	57.84	27.64	0.00–100
	CWF	16.18	6.32	6–39	7	6.88	4.62	0–24
	MCA	9.14	1.99	4–12	7	5.86	3.02	0–11
**Storytelling narrative**	Token counts	42.43	18.03	10–102	—	42.82	35.64	5–232
	Duration	35.85	15.80	7–79	—	84.54	67.53	11–359
	%CIU	83.14	12.87	53.13–100	64	57.67	29.11	3.70–100
	CWF	17.48	7.77	6–39	6	5.64	5.37	0–23
	MCA	6.70	1.95	2–9	4	2.60	1.88	0–9
**Procedural discourse**	Token counts	36.88	19.55	11–94	—	34.64	25.70	7–142
	Duration	21.54	11.37	5–56	—	59.86	48.02	8–190
	%CIU	86.56	11.94	51.22–100	69	64.06	27.57	8.00–100
	CWF	15.42	6.87	6–35	6	11.98	7.98	0–40
	MCA	9.90	1.55	5–12	8	6.24	3.49	0–12

*Note*: Duration measured in number of seconds. CIU, CWF, and MCA were significantly different between the two groups across all discourse tasks (*p* < 0.001).

Abbreviations: CIU = correct information units, CWF = content word fluency, MCA = main concepts analysis.

*Impaired if scored < cut‐off score.

**FIGURE 2:**
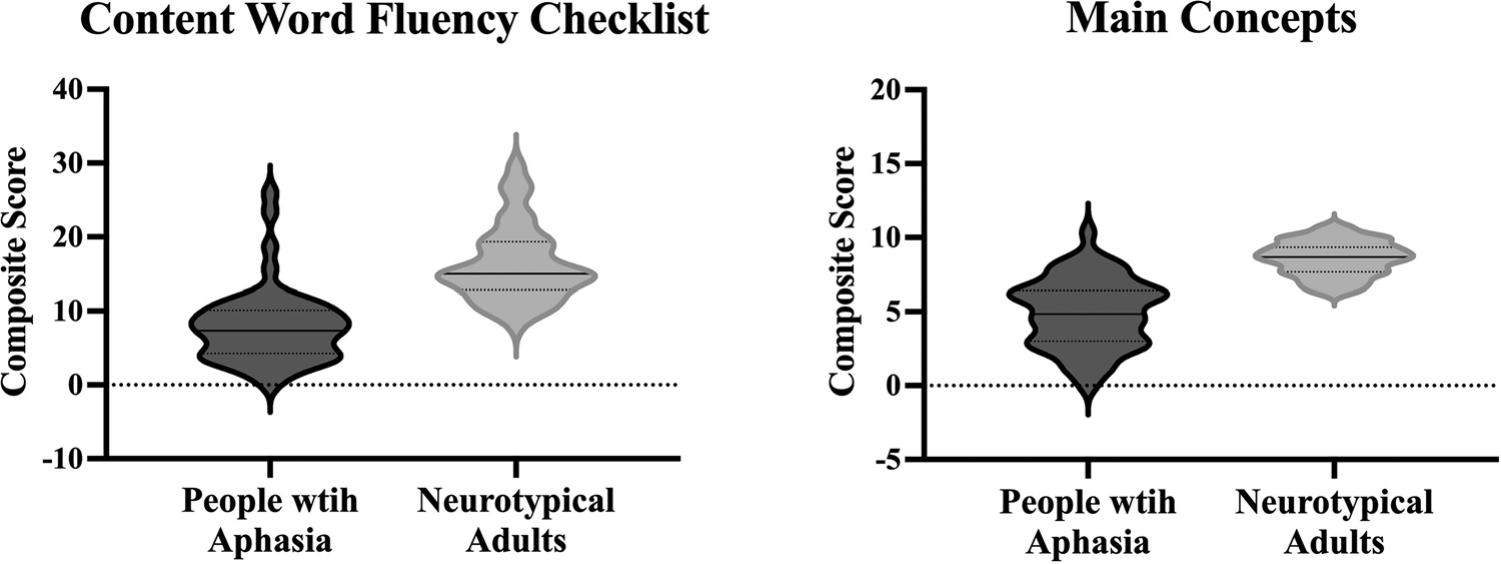
Comparison between the performances of neurotypical control group and aphasia group on transcription‐less discourse assessment approaches. Violin plots showing the distribution of data and the probability density of content word fluency (CWF) checklists and main concept analysis (MCA) during spoken discourse in groups of neurotypical controls (*N* = 70) and people with aphasia (*N* = 50). Straight lines refer to the group median, top dotted lines refer to the third quartile, and bottom dotted lines refer to the first quartile. The differences between the two groups on both approaches was statistically significant at *p* < 0.001.

One‐way ANOVA showed a significant difference between the two groups on the CIU (*F* (1, 118) = 21.71, *p* < 0.001, *η^2^* = 0.15), with higher scores obtained by the neurotypical control group (mean = 103.65, SD = 36.58) compared to the aphasia group (mean = 67.76, SD = 47.44). Results from one‐way ANOVAs that were conducted to determine if the newly developed approaches (CWF and MCA) could distinguish spoken discourse produced by PWA from that of neurotypical control speakers revealed significant differences between the two groups on the CWF approach (*F* (1, 118) = 76.88, *p* < 0.001, *η^2^* = 0.4) and MCA (*F* (1, 118) = 129.83, *p* < 0.001, *η^2^* = 0.53) with higher composite scores obtained by the neurotypical control group on CWF (mean = 48.81, SD = 15.55) compared to the aphasia group (mean = 23.7, SD = 15.23) and on MCA (mean = 25.74, SD = 3.46) compared to the aphasia group (mean = 14.7, SD = 6.95). The performance of the two groups is illustrated in Figure 2.

## 4 Discussion

To our knowledge, this is the first study to both administer transcription‐less, efficient discourse assessment approaches in real‐time during clinical examination to assess deficits in spoken discourse in PWA and to develop and validate both CWF checklists and MC lists in a non‐English speaking group. Evidence from this study suggests that these two transcription‐less approaches can be derived from discourse samples of neurotypical individuals and effectively applied in real‐time clinical settings with PWA. Moreover, findings indicate that these approaches can be adapted and validated for use in different languages, including Arabic, to allow accurate and efficient assessments of spoken discourse in relation to content word production and accuracy/completeness of information that are relevant to the topic under discussion during spoken discourse. This underscores the utility of such approaches to enhance the clinical assessment of expressive language skills across diverse and complex linguistic contexts.

### 4.1 Transcription‐Less Assessments of Spoken Discourse

The utilisation of discourse tasks has garnered increasing interest as a means to comprehensively and accurately assess expressive language abilities. This interest stems from recognition that relying solely on single‐word naming, a common practice in aphasia clinical examinations, may not fully capture the individual's true language proficiency in more naturalistic contexts (Alyahya et al. 2022; Stark 2019). However, integrating discourse tasks into clinical practice presents challenges due to their time‐intensive administration and scoring requirements. Consequently, there has been a concerted effort to develop accurate and efficient transcription‐less approaches for analysing discourse responses, including MCA (Nicholas and Brookshire 1993, 1995) and CWF (Alyahya et al. 2022). Not only is the CWF approach easy to apply and time efficient, but crucially, scores on this approach highly are correlated with the resultant outcome from the traditional, time‐consuming transcription and quantitative measures, demonstrating high ecological validity. Furthermore, scores on the Arabic CWF checklists and MC lists could differentiate the performance of PWA from neurotypical adults, demonstrating high construct validity. These approaches offer promising alternatives to traditional transcription‐based methods, allowing for quick and precise analysis of discourse without the need for transcription or detailed analyses.

For three discourse tasks (descriptive, storytelling, and procedural), Arabic MC lists and CWF checklists were developed using neurotypical's data. MC lists assess the presence of essential discourse elements, the accuracy of the provided information, and the completeness of essential discourse information. On the other hand, CWF is used to assess the production of content words during spoken discourse in PWA. The procedures used here to develop CWF checklists and MC lists replicated the original studies (Alyahya et al. 2022; Kong 2009; Nicholas and Brookshire 1995) but on speakers of a different language (Arabic). The use of these Arabic discourse assessment approaches can replace the current problematic practice of using informal assessments or non‐adapted English assessment tests with Arabic speakers with aphasia (Khoja 2019), which can result in inaccurate diagnosis and management (Altaib et al. 2021; Khoja 2019) due to linguistic and cultural differences. Specifically, the two approaches can be used to diagnose the presence of discourse deficits in PWA, using the norms and cut‐off scores provided in Table 3. This is to determine deficits in the fluency of content words (microlinguistics) and the use of accurate and complete concepts (macrolinguistics) during spoken discourse. These accurate diagnoses can guide treatment planning in PWA. Moreover, the Arabic CWF and MCA can be used as outcome measurements during clinical trials. This will have a remarkable impact on the quality of clinical management and research for PWA in Arabic countries, as well as Arabic speakers and bilingual speaking (e.g., English‐Arabic and French‐Arabic) immigrants living in Western countries.

### 4.2 Discourse Performance in PWA

The findings from this study revealed that Arabic PWA were impaired in both the production of content words and the accuracy and completeness of relevant information during spoken discourse, compared to neurotypical adults. These findings resonate with results on English speakers, where PWA produced significantly fewer content words during picture description and storytelling narratives (Alyahya et al. 2022) and retrieved fewer words during picture‐sequence description (Dalton and Richardson 2015; Kim et al. 2019) compared to neurotypical adults. Although these studies used different discourse stimuli and participants spoke different languages, they all found deficits in word retrieval and content word production during spoken discourse in aphasia. The observed discrepancy between PWA and neurotypical adults in the production of content words during spoken discourse highlights a fundamental difference in language proficiency. Content words, such as nouns, verbs, adjectives, and adverbs, are crucial elements for conveying meaning and substance in communication. PWA typically exhibit impairments that can significantly impact their ability to retrieve and produce content words effectively (Alyahya et al. 2021; Kim et al. 2019; Stark 2019). These impairments may stem from underlying difficulties with lexical access, semantic processing, and/or syntactic organization (Alyahya 2024b; Foygel and Dell 2000). As a result, PWA often struggle to generate a diverse and rich vocabulary during spoken discourse, leading to a reduced overall production of spoken discourse. In contrast, the ability of neurotypical adults to access and retrieve lexical items quickly and accurately facilitates fluent and coherent communication, enabling them to convey ideas and information more effectively (Alyahya et al. 2022; Dalton and Richardson 2015).

In this study, PWA produced less key information than neurotypical controls across different discourse tasks (descriptive, narrative and procedural). Although most MCs were correctly and accurately produced by neurotypical adults, they were mostly absent by PWA. This is similar to findings from previous studies in other languages, including English (Dalton and Richardson 2015) and Cantonese (Kong 2009). One possible explanation for the difference between PWA and neurotypical adults is the linguistic impaired characteristic of aphasia, which can manifest as difficulties with word retrieval, sentence construction, and discourse organisation. As a result, individuals with aphasia may struggle to generate and convey the necessary content to communicate their ideas and experiences effectively. The absence of correctly and accurately produced key concepts by PWA further highlights the impact of aphasia on their ability to convey essential information during communicative interactions effectively.

These findings suggest that PWA may struggle not only with producing sufficient content words (as measured by CWF) but also with accurately generating and organising key concepts within their discourse (as measured by MCA). The discrepancy between PWA and neurotypical adults in producing content words and key information underscores the importance of considering the quality and accuracy of language output in addition to quantity. While neurotypical adults may produce more information, it is crucial to assess the accuracy and relevance of this information. In contrast, PWA may exhibit deficits in both quantity and quality, leading to reduced spoken discourse. Understanding the underlying mechanisms contributing to reduced content word production and key discourse information in aphasia can inform the development of innovative treatment approaches and assistive technologies tailored to the specific needs of PWA.

Overall, these findings endorse the importance of considering both quantity and quality metrics when assessing language abilities in clinical and research settings (Alyahya et al. 2020). Additionally, they emphasise the need for tailored assessment approaches to address the unique communication challenges faced by PWA. By understanding the specific nature of language deficits associated with aphasia, clinicians and researchers can develop targeted strategies to improve communication outcomes and enhance the quality of life for individuals with aphasia.

### 4.3 Limitations and Future Directions

Test‐retest reliability of the two approaches (CWF and MCA) could not be conducted in this study, as all participants were examined in one session. Furthermore, the study did not compare discourse measures to each other. An avenue of future research is to examine whether performance on MC and CWF differs between different discourse tasks. This is important, as previous studies revealed that the type of discourse stimulus can influence the linguistic elements of spoken discourse (Alyahya et al. 2020; Alyahya et al., 2022; Schnur and Wang 2024). Comparing discourse measures would be interesting in determining which measures would best distinguish discourse responses produced by PWA from those produced by neurotypical adults. Such findings would discern the best discourse approach to implement in clinical settings. For example, comparing the validity and reliability of CWF to those of core‐lexicon approach would be an interesting focus of future research. It would also be interesting to explore the attitudes and experiences of SLTs with the use of CWF and MCA during clinical examination.

To examine the diagnostic sensitivity of these discourse approaches, their validity should also be examined using a representative sample of the population under study (Rohde et al. 2018); that is, can these approaches differentiate discourse responses produced by PWA from the responses by another control group of people who have survived brain damage or neurological condition but without any language impairment.

### 4.4 Clinical Implications

The transcription‐less, efficient, and accurate discourse assessment approaches developed, validated, and examined in this study provide unique and objective resources for clinicians providing services to Arabic speakers with aphasia. The CWF checklists and MC lists are provided in the Supporting Information, including scoring sheets and instructions. Moreover, the successful and reliable application of these approaches (CWF and MCA) in real‐time clinical settings during clinical examinations demonstrates the immense potential these approaches have in enhancing clinical practice, as it provides assurance to SLTs that it is viable to use these approaches during the session without the need to record discourse responses or transcribe, analyse and score them offline after the session has ended. Indeed, these approaches are easy to administer and do not require advanced linguistic skills and thus have the potential to be used by all healthcare professionals (e.g., neurologists, psychiatrists, and nurses) to screen for expressive language deficits beyond word and sentence levels following brain damage and neurological conditions.

The novel discourse approaches developed in this study will encourage SLTs to incorporate discourse tasks as part of their routine clinical examination to be used (i) during initial examination for a comprehensive assessment of expressive language skills at higher levels and beyond words and sentences, (ii) to guide the development of personalised therapy goals and strategies to improve communication in PWA, and (iii) as outcome measurements to monitor changes, either from spontaneous recovery or improvements in response to interventions. Furthermore, these approaches have the potential to be used in research as part of a core set of discourse outcome measures (Dietz and Boyle 2017).

## 5 Conclusions

Two valid and reliable transcription‐less, efficient, and clinically friendly discourse assessment approaches (CWF and MCA) have been developed and validated in Arabic. Furthermore, the two approaches have been applied and examined in real‐time clinical settings with PWA. The evidence from the current study endorses the incorporation of discourse tasks into the routine clinical examination of aphasia. This can be achieved through the utilisation of valid, time‐efficient, and accurate approaches to assess deficits in the production of content words (using CWF checklists) and information accuracy/completeness (using MCA) of spoken discourse. These approaches allow clinicians to score discourse responses during clinical examinations or research sessions without the need for transcription, analysis or coding. This meets the goal of ‘transcription‐less discourse sampling’, which has been described previously as ‘a clinician's dream’ (L. Armstrong et al. 2007).

## Ethics approval statement

This study was approved by King Fahad Medical City's Institutional Review Board (IRB No. 20‐763).

## Consent statement

All participants provided written informed consent before voluntarily participating in this study according to the Declaration of Helsinki under the approval of the local research ethics committee.

## Supporting information



Supporting Information

## Data Availability

The data that support the findings of this study are available on request from the corresponding author. The data are not publicly available due to privacy or ethical restrictions.
