# Supplementary material for: Development and Real‐Time Clinical Application of New Transcription‐Less Discourse Assessment Approaches for Arabic Speakers With Aphasia
Source: Int J Lang Commun Disord. 2025 May 5;60(3):e70043. doi: 10.1111/1460-6984.70043 (PMC12051822; doi:10.1111/1460-6984.70043)
Supplement: Supplementary file 1 — Supporting Information [file JLCD-60-0-s001.pdf]

Arabic Discourse Assessment Tool (ADAT)

Dr Reem Alyahya

IJLCD
2025

Efficient and clinically-friendly discourse assessment tools

Scoring sheets

Content Word Fluency (CWF):

English translation	Target item	Acceptable alternative items	# of produced times
**Lounge - composite picture description**			
Men	رجال	ضيوف - مجموعة - أهل- اخوان – أخويه - عائلة - جماعة - ناس - أشخاص - شباب	
Sitting	جالسين	يتقهون – متعازمين – متربعين – متكين – قاعدين	
In	في		
Lounge/Living Room	مجلس	بيت استراحة – منزل – صالة – اجتماع – ضيافة – جلسة – جمعة	
Man	رجل	صاحب - أحد – انسان – شاب – شخص	
Smoking	يدخن		
Pouring	يصب	يسكب - يكب	
Coffee	قهوه	يقهوي	
Child	طفله	بزر - بنت	
Slipped	زلقت	تتزحلق - تصدم – تعثرت – تطيح – تسقط – طاحت	
Car	سيارة	ألعاب	
		**Total Score**	
**Kitchen – storytelling narrative**			
Lady	مرأه	حرمه - سيدة - أم – زوجة	
Cooking	تطبخ	تصلح - تعمل -تجهز – تحضر - تعد - تسوي – تطهي	
In	في		
Kitchen	مطبخ	منزل - بيت	
Tasting	تتذوق	تأكل	
And	و		
Talking	تتكلم	ترد - تخاطب – تتحدث – تتصل – تسولف	
Phone	تلفون	جوال – هاتف - كلام - محادثة –مكالمة	
Burned	أحترق	يتبخر - فاح – نست – حطت – يغلي	
Food	أكل	قدر – وجبة – غدا - عشاء - طعام - طبخه	
		**Total Score**	
**Tea – procedural discourse**			
Boil	أغلي		
Water	مويه	ماء	
Then	بعدين	ثم	
Put	أحط	أضيف - أعبي - أضع	
Tea	شاهي	لبتون - تلقيمه – شاي	
And	و		
Sugar	سكر		
On	على		
Stove	نار	غلاية – بوتقاز – غاز	
Pour	أصب	أكب – أسكب	
In	في		
Teapot	ابريق	براده - ترمس	
		**Total Score**	

Scoring instruction for CWF:

- Each discourse stimuli is scored separately.
- One point is assigned every time the speaker produced one of the target items or an acceptable alternative item (including when the target items are used again in different phrases) but excluding immediate repetitions and perseverations.
- Variations in word form are acceptable, e.g., 'يجلسون' – 'يجلس' – 'جالسين' for 'جالس'.
- Target items with bound morphemes are acceptable, e.g., 'أحطها' – 'تحطين' for 'أحط'.
- **Total score** = sum of all the points.
- **Deficits** in content word fluency during spoken discourse is deemed if the total score is below the following cut-off norms:
  - Lounge - composite picture description task ≤ 7
  - Kitchen – storytelling narrative task ≤ 6
  - Tea – procedural discourse task ≤ 6

Main concept Analysis (MCA):

English translation	Main concepts	Response				
		AC	AI	IC	II	AB
**Lounge - composite picture description**						
Men **sitting** in the lounge	رجال **جالسين** في المجلس					
Girl **fell** on the floor	بنت **طاحت** على الأرض					
The man is **pouring** coffee for the guests	الرجال **يصب** القهوه للضيوف					
The man is **smoking** outside by the window	الرجال **يدخن** برا عند الشباك					
	**Total Score**					
**Kitchen – storytelling narrative**						
The woman is **cooking** the food in the kitchen	المرأة **تطبخ** الأكل بالمطبخ					
The woman is **speaking** on the phone	المرأه **تكلم** بالتلفون					
The woman **forgot** the food on the stove	المرأه **نست** الأكل على النار					
	**Total Score**					
**Tea – procedural discourse**						
I **boil** the water in the kettle	**أغلي** الموية بالغلاية					
After the water **boils**	بعد ما **تغلي** الموية					
I **pour** the tea in the teapot	**أصب** الشاهي بالابريق					
And I **add** sugar	و **أحط** السكر					
	**Total Score**					

Scoring instruction for MCA:

- Each concept is given one of the following five codes based on the completeness and accuracy of the essential elements (underlined items):
  - AC: if the speaker produced all essential elements correctly.
  - AI: if the speaker omitted at least one essential element, but correctly produced the other elements.
  - IC: if the speaker produced all essential elements, but at least one essential element was inaccurate.
  - II: if the speaker produced at least one essential element incorrectly, plus they omitted at least another essential element.
  - AB: if the speaker did not produce any essential element.

- Acceptable alternative items are similar to the ones provided in CWF table.
- Scoring is completed as follow:
  - Each AC is given 3 points
  - Each AI is given 2 points
  - Each IC is given 2 points
  - Each II is given 1 point
- **Total score** = sum of all the points.
- **Deficit** in the accuracy and completeness of key concepts in spoken discourse is deemed if the total score is below the following cut-off norms:

- Lounge - composite picture description task ≤ 7
- Kitchen – storytelling narrative task ≤ 4
- Tea – procedural discourse task ≤ 8

References:

- Alyahya, R. S. W. (2024). The development of a novel, standardized, norm-referenced Arabic Discourse Assessment Tool (ADAT), including an examination of psychometric properties of discourse measures in aphasia. *International Journal of Language & Communication Disorders,* 59(5), pp. 2103-2117. doi: 10.1111/1460-6984.13083

- Alyahya, R. S. W. (2025). Development and Real-Time Clinical Application of New Transcription-Less Discourse Assessment Approaches for Arabic Speakers with Aphasia. *International Journal of Language & Communication Disorders*. doi: 10.1111/1460-6984-70043

Discourse Stimuli

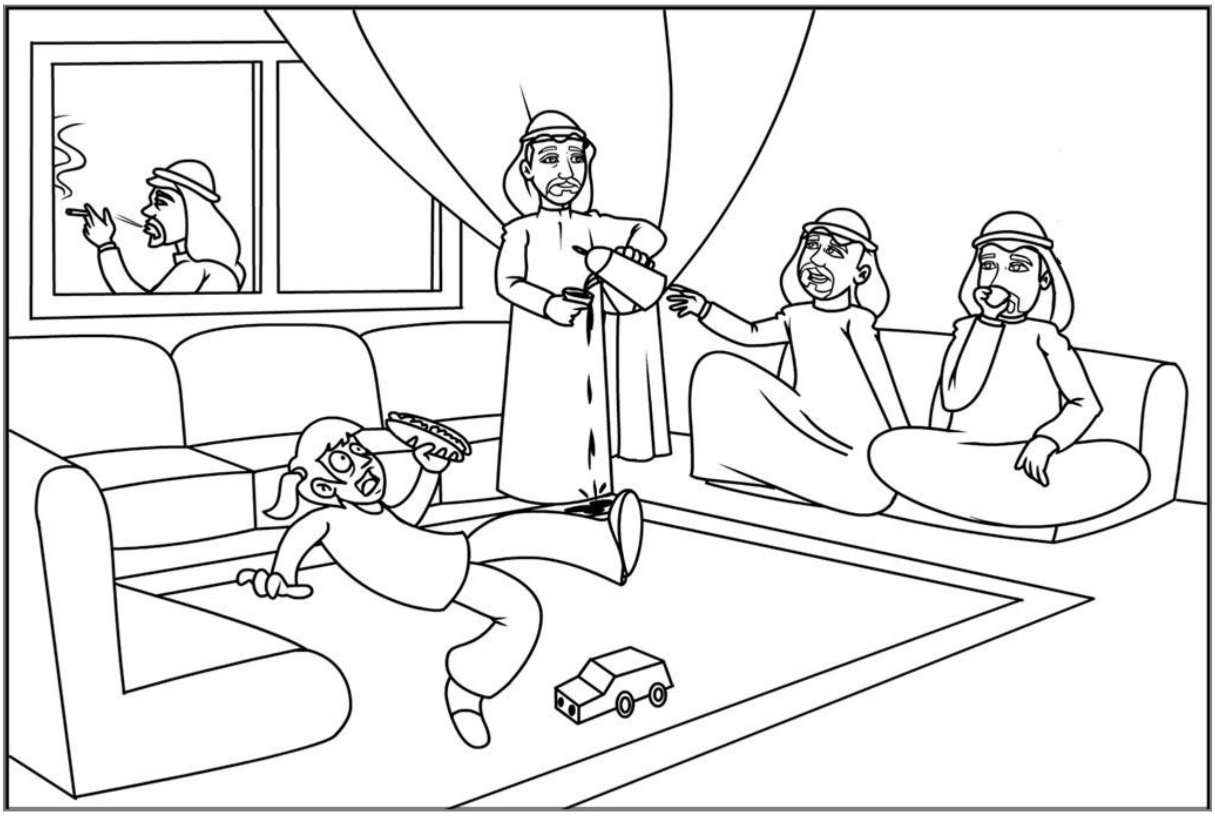

'Lounge' composite picture description.

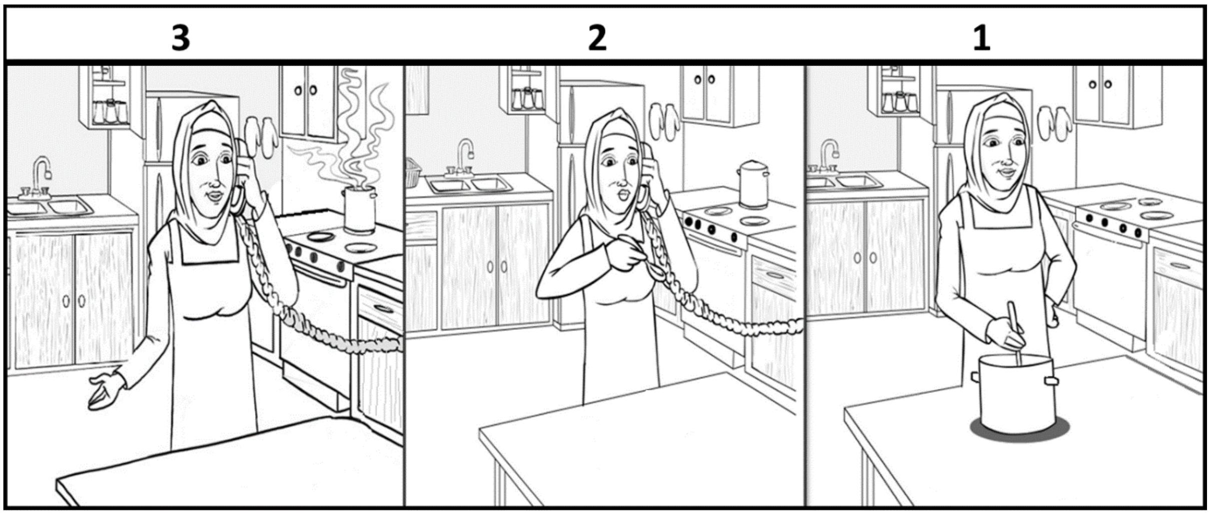

'Kitchen' storytelling narrative.
